# Interactive and Play-Based Group Education Is Associated with Improvements in Carbohydrate Counting Skills and Self-Care Confidence in Children and Adolescents with Type 1 Diabetes: An Exploratory Study

**DOI:** 10.3390/nu18050790

**Published:** 2026-02-27

**Authors:** Sabine Schade Jacobsen, Zandra Overgaard Pedersen, Emilie Nyholm-Christensen, Bettina Ewers

**Affiliations:** Steno Diabetes Center Copenhagen, Department of Diabetes Care, Copenhagen University Hospital, Borgmester Ib Juuls Vej 83, 2730 Herlev, Denmark; zandra.overgaard.pedersen@regionh.dk (Z.O.P.); emilie.nyholm-christensen@regionh.dk (E.N.-C.)

**Keywords:** carbohydrate counting, type 1 diabetes (T1D), children, adolescents, group-based education, self-care, dietitian, self-confidence

## Abstract

**Background/Objectives:** Effective glycemic management from the time of diagnosis is essential in the care of children and adolescents with type 1 diabetes (T1D), as early glycemic patterns can influence long-term health outcomes. **Methods**: This exploratory study evaluated a one-month interactive, group- and play-based education program designed to enhance food and carbohydrate counting skills among families of children and adolescents with newly diagnosed (ND) T1D (<1 year since diagnosis) or suboptimal glycemic control (SGC) (hemoglobin A1c (HbA1c) > 7.5% (58 mmol/mol)). The intervention included hands-on learning activities in food and carbohydrate counting, and peer interaction to support development of diabetes self-management skills. Data were collected at baseline, post-intervention, and at six-months follow-up through medical records, glucose sensor data, and a questionnaire assessing diabetes self-management skills, dietary practices, and carbohydrate counting. **Results:** Between September 2022 and April 2024, 55 children and adolescents were enrolled in the ND group and 22 in the SGC group. Post-intervention, carbohydrate counting skills improved, particularly in the ND group. Participants reported greater confidence and independence in carbohydrate counting and insulin dosing, with parents noting sustained benefits at six-months follow-up. No significant changes were observed in glycemic control, including time-in-range and postprandial glucose profiles. **Conclusions**: In this exploratory study, early interactive and play-based group education was associated with improvements in carbohydrate counting skills and self-care confidence in children and adolescents with newly diagnosed T1D. These improvements were not accompanied by changes in glycemic outcomes. The findings occurred during a complex and transitional phase following diagnosis. Further research is needed to examine sustainability and long-term clinical impact.

## 1. Introduction

Type 1 diabetes (T1D) is a chronic autoimmune disease characterized by immune-mediated destruction of pancreatic β-cells, leading to absolute insulin deficiency and lifelong insulin dependence [1]. The global incidence of T1D among children and adolescents is increasing, with marked geographic variation. In Denmark, approximately 300 new pediatric cases are diagnosed annually, with an estimated yearly increase of about 3% [2,3]. Establishing effective glycemic management from the time of diagnosis is a key priority in T1D care. Research indicates that glycemic control established early after T1D diagnosis is associated with a lower risk of long-term complications and mortality [4,5,6]. This association is particularly evident in children and adolescents: those with elevated hemoglobin A1c (HbA1c) levels (≥8.6% (70 mmol/mol)) during the first years after diagnosis tend to maintain higher HbA1c levels into adulthood compared to those who achieve good glycemic control (HbA1c < 6.7% (50 mmol/mol)) early on [7,8,9]. However, achieving and maintaining optimal glycemic control is complex and influenced by multiple factors, with diet playing a pivotal role [10]. Several studies have demonstrated that family-supported adherence to a healthy diet and accurate mealtime insulin dosing are associated with improved glycemic control and quality of life in children and adolescents with T1D [11,12].

Carbohydrate counting is a well-established, evidence-based approach to mealtime insulin dosing and a central component in achieving glycemic control in children and adolescents with T1D [13,14,15,16,17]. However, studies indicate that both children and adolescents with T1D, as well as their parents, often misestimate carbohydrate content of foods, leading to incorrect mealtime insulin dosing and poorer glycemic control [13,18]. In addition, living with T1D can be challenging, as it requires constant attention to healthy eating, carbohydrate counting, meal planning, and the dosing and timing of insulin. This ongoing focus may contribute to a strained relationship with food and an increased risk of eating disorders in individuals with T1D [19]. However, recent evidence suggests that individuals with T1D who use carbohydrate counting may be less likely to exhibit signs of disordered eating, although its potential role requires further investigation [20].

To promote healthy eating, ensure adequate knowledge and skills in carbohydrate counting and mealtime insulin dosing, and at the same time foster a positive relationship with food, international clinical guidelines recommend that children and adolescents with T1D, along with their families, receive regular dietary counseling from a registered dietitian. This is considered an essential component of diabetes management, both at diagnosis and as ongoing support to reinforce skills [19,21,22,23].

The International Society for Pediatric and Adolescent Diabetes (ISPAD) guidelines highlight the benefits of group-based, collaborative education, emphasizing the importance of involving the entire family in decision-making and implementing appropriate changes [23]. Studies have shown that structured group education in carbohydrate counting can improve HbA1c levels and overall well-being in adults with T1D compared with standard care [24]. A study in 2016 investigated the impact of a five-day group education program for children with T1D, led by a nurse and a dietitian, which included training in carbohydrate counting. The results showed improved quality of life among children who participated in the structured program compared with those receiving standard care. The program also received highly positive feedback from parents [25].

Accordingly, there was a need to develop and explore a dietitian-delivered, group-based dietary intervention incorporating a hands-on, play-based approach to food and carbohydrate counting for families of children and adolescents with T1D in a Danish context. Such an approach may foster a more relaxed and positive relationship with food, while strengthening self-care skills and confidence among families. This applies both to newly diagnosed children and adolescents and those living with T1D who have not achieved glycemic targets. The aim of this study was to examine potential changes in self-care skills (including carbohydrate counting and insulin management), glycemic outcomes, and emotional well-being associated with participation in an interactive, group- and play-based education program focusing on food and carbohydrate counting for families of children and adolescents with newly diagnosed T1D or suboptimal glycemic control.

## 2. Materials and Methods

### 2.1. Design and Participants

This study is an exploratory, single-arm pre–post study with a one-month intervention period followed by six-months of follow-up. Between September 2022 and April 2024, children and adolescents with newly diagnosed T1D admitted to Herlev Hospital for initial stabilization and subsequently referred to the outpatient diabetes clinic at Steno Diabetes Center Copenhagen (SDCC) in the Capital Region of Denmark were invited to participate in an education program within the first year after diagnosis. The study follows the guidelines for reporting non-controlled studies [26].

Inclusion criteria were children and adolescents aged 0–15 years with T1D treated at SDCC who were either newly diagnosed (ND) (<1 year since diagnosis) or had suboptimal glycemic control (SGC), defined as HbA1c > 7.5% (58 mmol/mol). Exclusion criteria included inability to receive education in Danish, inability to understand the informed consent and study procedures, or concomitant participation in other trials.

Participants were recruited through healthcare professionals at Herlev Hospital and SDCC who were involved in their treatment, as well as through SDCC’s website, and informational materials displayed in the clinic’s waiting areas.

The education program was developed based on a program theory assuming that strengthening diabetes-related self-care competencies would support improvements in glycemic control. Accordingly, the program was designed to enhance practical skills in meal planning, carbohydrate counting, mealtime insulin dosing, and blood glucose management through interactive, age-adapted, and supportive learning activities. In addition to skill development, the group-based format aimed to promote emotional well-being, reduce stress related to dietary management, and strengthen confidence in everyday diabetes self-management. Within this framework, improvements in glycemic control were considered a clinically relevant outcome, expected to reflect strengthened self-care practices and increased competence in managing food and insulin in daily life. Participating parents were likewise expected to perceive greater confidence and competence in their child’s diabetes management over time.

The primary outcomes were changes in glycemic control (measured as time-in-range (TIR)) from baseline to post-intervention (one month) and six-month follow-up among participating children and adolescents in the ND and SGC group.

Secondary outcomes included changes in additional glycemic metrics derived from continuous glucose sensor data (postprandial glucose excursions, time-above-range (TAR), time-below-range (TBR), coefficient of variation (CV), and estimated HbA1c), as well as self-care skills (including dietary practices, carbohydrate counting, insulin mealtime dosing, and blood glucose management), and emotional well-being from baseline to post-intervention (one month) and six-month follow-up among participating children and adolescents in the ND and SGC group. Additionally, the study aimed to assess changes in parents’ perceptions of their child’s confidence and competence in diabetes management (particularly in carbohydrate counting and insulin dosing) from baseline to post-intervention (one month) and six-month follow-up.

### 2.2. Education Program

The education program was offered to two groups: (1) children and adolescents with newly diagnosed T1D, defined as <365 days from diagnosis (Newly Diagnosed, ND group) and (2) those with suboptimal glycemic control, defined as HbA1c > 7.5% (58 mmol/mol) (Suboptimal Glycemic Control, SGC group). For the SCG group, the educational sessions were organized into three age-specific groups: 0–5, 6–10, and 11–15 years. In contrast, the ND group was not subdivided by age, as the primary focus was to deliver the intervention during the early post-diagnosis period rather than to differentiate by age. All participants were encouraged to attend with a parent or family member.

**Figure 1:**
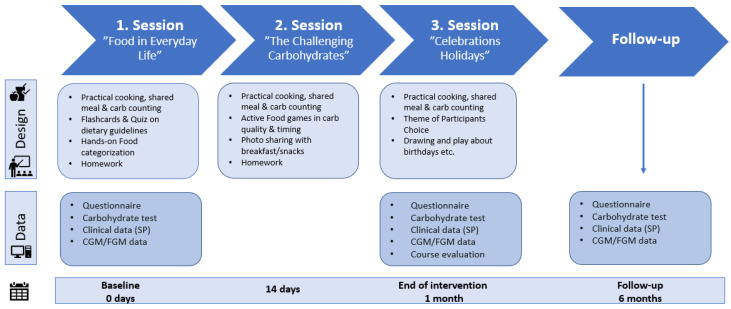
Study design, timeline, and data collection. Abbreviations: CGM, continuous glucose monitoring; FGM, flash glucose monitoring; SP, Sundhedsplatformen (electronic medical journal system).

The structure and content of the education program are presented in Figure 1. The program was a one-month intervention consisting of three sessions, each lasting two hours, and structured around the following themes: “Food in Everyday Life”, “The Challenging Carbohydrates” and “Special Occasions, Celebrations and Holidays”. Each session incorporated interactive, hands-on, play-based learning activities, including exercises in carbohydrate counting, cooking classes, and instruction on food and health in relation to T1D. The health education-based approach was designed to be playful and engaging, emphasizing interactive exercises, peer-to-peer learning activities, and physical movement. Sessions were held in the educational kitchen “FoodLab” at SDCC and facilitated by nutrition specialists and registered dietitians.

### 2.3. Data Collection and Management

Three types of data were collected at three time points: (1) at baseline (before the first session of the educational program), (2) at end-of-intervention (upon completion of the program), and (3) at six-months follow-up.

#### 2.3.1. Medical Data

Information was retrieved from the participants’ electronic medical records using “Sundhedsplatformen”: age, gender, diabetes duration, most recent HbA1c level, body weight and height.

#### 2.3.2. Glucose Sensor Data

Fourteen days of historical glucose data were extracted from participants’ electronic devices, including flash glucose monitoring ([FGM], the Freestyle Libre systems (1 and 2), Abbott Diabetes Care, Alameda, CA, USA) continuous glucose monitoring ([CGM], The Dexcom sensor systems (G6 and G7), Dexcom, SD, CA, USA; Guardian sensor system (3 and 4), Medtronic, Minneapolis, MN, USA) and/or insulin pumps (The Tandem insulin pump system, Tandem Diabetes Care, SD, CA, USA; The Medtronic insulin pump systems (670G and 780G), Minneapolis, MN, USA; The Omnipod insulin pump systems (Omnipod Dash and Omnipod 5), Insulet Corporation, MA, USA). Data were obtained using upload programs such as Stenopool (Line Systems Aps, CPH, DK and Steno Diabetes Center Copenhagen, Herlev, Denmark, version 1.17.4), LibreView (Abbott Diabetes Care, Alameda, CA, USA, version 1.0 and 2.1), Glooko (Glooko Inc., Palo Alto, CA, USA, version 22.2—22.5), and CareLink (Medtronic, Minneapolis, MN, USA, version 2.3.1—3.3.0). Due to technical difficulties or compatibility issues with some devices or software, complete data could not be retrieved for all participants.

#### 2.3.3. Questionnaires

Participants completed study-specific questionnaires administered either by email though the REDCap system (Vanderbilt University, Nashville, TN, USA, version 12.5.9—14.0), approved for data collection and storage in the Capital Region of Denmark, or in paper form after the final education session. It was not possible to receive six-months follow-up questionnaires in paper form. The questionnaires used were not validated, as no existing instruments were found to adequately address the study’s aims for this specific population. Therefore, the items were developed based on elements from existing pediatric quality of life measures, particularly the Pediatric Quality of Life Inventory version 3 [27] and adapted to incorporate dietary context. The questionnaires were pilot-tested in a small sample from the target population to ensure comprehension. Items were grouped into the following themes: diabetes management, dietary practice, and carbohydrate counting. Responses from participants under the age of five were excluded from analysis due to concerns about question comprehension [28].

Two versions of questionnaires were developed: one for children and one for parents. The children’s version included smiley-face response options to enhance engagement and comprehension, and younger children were guided through the questionnaire. For a complete list of questionnaire items, see Supplementary File S1 and S2.

A questionnaire to assess carbohydrate estimation accuracy skills was also administered. This questionnaire included 12 images showing various portion sizes of different food items (candy, lasagna, potato wedges, pasta, pizza slice, rice, fruit tart, milk chocolate, oatmeal, raisins, cantaloupe melon, and a whole-grain bun; see Supplementary File S3). Participants were asked to independently estimate the carbohydrate content of each portion without any assistance. This carbohydrate estimation questionnaire has previously been validated [29].

In addition to the data described above, participants completed an anonymous evaluation form at the end of the program to assess their experience with the education course (See Supplementary File S4). Questionnaire responses collected using a 5-point Likert scale were analyzed using paired *t*-tests.

### 2.4. Ethics

According to the Scientific Ethics Committees of the Capital Region of Denmark, this health science intervention study did not require ethical approval (journal no.: H-23018075). Written informed consent was obtained from the parents or legal guardians of the participating children and adolescents prior to study inclusion. The study was approved for data storage by the Danish Data Protection Agency (journal no.: P-2023-272).

### 2.5. Data Analysis

Baseline characteristics are reported as means with standard deviations (SD) for normally distributed continuous variables, medians with interquartile range (IQR) for non-normally distributed continuous variables and as frequencies with percentages for categorical variables.

For the carbohydrate estimation questionnaire, participants’ estimated carbohydrate content for each of the 12 food items and these were compared with the correct values, and delta values (estimation errors) were calculated. Delta values for estimation errors were calculated by comparing baseline estimates with those at the end of the one-month intervention and at six-month follow-up. To assess changes in estimation accuracy over time, the absolute estimation error for the same participant and for each item was compared across the two time points. Participants were categorized into three groups based on their overall estimation performance: (1) “Improved”—if accuracy improved for the majority of food items; (2) “Worsened”—if accuracy declined for most food items; and (3) “Unchanged”—if there was no change in accuracy across items or an equal part was improved or worsened. Results are presented as the proportion of participants classified as “Improved”, “Worsened”, or “Unchanged”. Mean delta values were not used for analysis, as large estimation errors in a single food item could disproportionately skew overall results.

Sensor data, including sensor glucose levels (TAR, TBR, TIR), CV, and estimated HbA1c, were analyzed using unpaired *t*-tests. An unpaired *t*-test was chosen in order to include as much data as possible, since data availability at six-month follow-up was low. Comparisons were made between baseline and end-of-intervention at one month, as well as between baseline and the six-month follow-up. Analyses were conducted both with and without adjustments for changes in insulin pump therapy. Sensor data was excluded for participants (*n* = 4) with less than 80% active sensor time, due to concerns about data reliability [30].

The analysis of postprandial blood glucose excursions was conducted using Python, version 3.11.9 (Python Software Foundation, Wilmington, DE, USA). Three days of sensor data were selected for analysis, extracted from the five-day period before the first education session (baseline) and a five-day period following the final session (end-of-intervention). This selection aimed to exclude weekend days. Area under the curve (AUC) values were calculated by first identifying meal start times, defined as a rise in blood glucose of at least 0.03 mmol/L per minute sustained for 30 min, indicating a meal-related plasma glucose rise, as previously described [31,32]. Meal start times were required to fall within predefined time intervals: breakfast (5:00 AM–10:00 AM); lunch (11:00 AM–2:00 PM); and dinner (5:00 PM–9:00 PM). For each identified meal, AUC was calculated from the meal start time to three hours post-meal using the trapezoidal rule. If multiple AUC values were generated for the same meal, the most appropriate value was manually selected. Mean AUC values were then calculated for breakfast, lunch, and dinner at each of the three time points: baseline, end of intervention, and at six-month follow-up. To assess differences over time, unpaired *t*-tests were used to compare AUC values for each meal type between baseline and the end of the intervention, as well as between baseline and the six-month follow-up.

## 3. Results

### 3.1. Study Population

Out of 191 children and adolescents (ages from 0 to 15 years) newly diagnosed with T1D between September 2022 and May 2024, 55 (29%) agreed to participate in the education program. Out of 344 children and adolescents with suboptimally controlled T1D (ages from 0 to 15 years), 22 (6%) accepted participation. The most common reasons for declining participation included lack of time, scheduling conflicts, and limited perceived need of dietary education. The overall mean age of all participants was 9.9 years, with a mean of 9.1 years in the ND group and 12.0 years in the SGC group. Baseline characteristics are presented shown in Table 1. Mean diabetes duration was 115 days in the ND group and 4.4 years in the SGC group. Nearly all participants used a glucose sensor, and 31% had an insulin pump at baseline.

### 3.2. Carbohydrate Estimation

**Figure 2:**
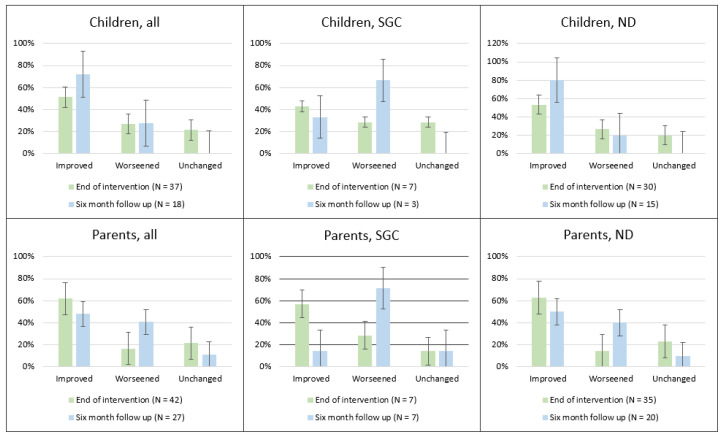
Changes in carbohydrate estimation accuracy skills at end-of-intervention after one month, and at six-month follow-up, compared with baseline. Abbreviations: Improved, if carbohydrate estimation accuracy improved for the majority of food items, compared with baseline data; ND, newly diagnosed diabetes; SGC, suboptimal glycemic control; unchanged, if there was no substantial change in carbohydrate estimation accuracy across food items, compared with baseline data; worsened, if carbohydrate estimation accuracy declined for most food items, compared with baseline data.

The categorization of participants based on changes in carbohydrate estimation accuracy skills—classified as improved, worsened, or unchanged—is presented in Figure 2. A high proportion of newly diagnosed children, adolescents, and their parents showed improvements after the education intervention, with many sustaining skills at the six-month follow-up. In contrast, fewer children, adolescents, and their parents in the suboptimal glycemic group showed improvements in carbohydrate estimation skills, with some even worsening over time.

### 3.3. Glucose Sensor Data Results

**Figure 3:**
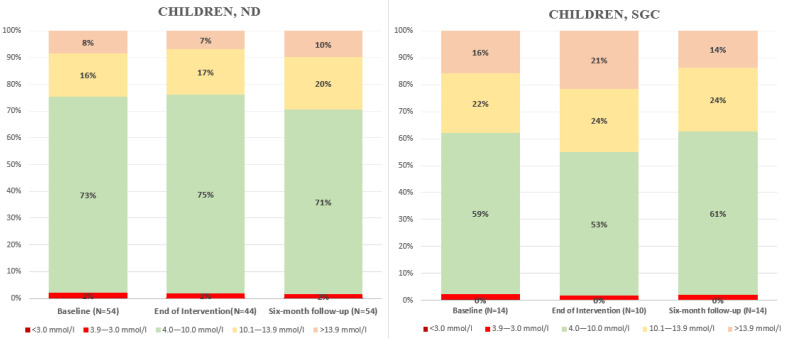
Glucose control based on sensor data at baseline, end-of-intervention after one month, and at six-month follow-up. Abbreviations: ND, newly diagnosed diabetes; SGC, suboptimal glycemic control.

No clinically relevant changes in glucose sensor data were found from baseline to end-of-intervention or at six-month follow-up. Figure 3 presents the data, with supplementary tables providing further details: Supplementary Table S5 shows data without insulin pump adjustments, while supplementary Table S6 includes adjustments for individuals who initiated insulin pump therapy during the intervention or follow-up period.

### 3.4. Postprandial Blood Glucose Fluctuations

No significant clinically relevant changes were observed in the AUC analyses. Mean AUC changes are presented in Supplementary Table S7.

### 3.5. Self-Reported Data on Everyday Life Changes

Table 2 and Table 3 present responses from children, adolescents, and parents to selected questions from the “Everyday Life” questionnaire. After the education intervention, more children and adolescents reported being able to count carbohydrates and dose mealtime insulin independently, and fewer found carbohydrate counting difficult. More parents similarly reported that their child could count carbohydrates independently. However, these perceived improvements were sustained only among parents at six-month follow-up. No significant changes were observed in the distribution of responses for the “Diabetes management” or “Dietary practice” domains. 

## 4. Discussion

This exploratory study examined changes in glycemic control, self-care skills, and emotional well-being associated with participation in an interactive, play-based group education program focusing on food and carbohydrate counting among children and adolescents with newly diagnosed T1D or suboptimal glycemic control and their families. The program was designed to strengthen diabetes self-management, particularly skills related to carbohydrate counting and mealtime insulin dosing.

Children, adolescents, and parents in the ND group demonstrated measurable improvements in carbohydrate counting accuracy, accompanied by self-reported gains in confidence and reduced perceived difficulty with carbohydrate counting tasks. These improvements suggest that the program helped families develop key components of diabetes self-care, including knowledge, skills, and confidence in managing diet and carbohydrate counting tasks during the early phase of living with T1D. While these improvements did not lead to detectable improvements in glycemic control, they represent important foundations for sustained diabetes self-care. Behavioral and educational interventions may influence clinical outcomes gradually, as knowledge acquisition and confidence-building often precede consistent behavioral implementation. It is possible that longer follow-up is required before measurable metabolic effects become apparent. Particularly in newly diagnosed children, the early post-diagnosis phase involves ongoing treatment adjustments and adaptation processes, which may obscure short-term glycemic changes. Similarly, no clinically relevant changes in glycemic outcomes were found in the SGC group. Although modest improvements in self-care skills and reported behaviors were noted, no corresponding changes in glycemic outcomes were observed. This may be related to the group’s longer diabetes duration and slightly older age, as established glycemic patterns and self-care or treatment routines may be more difficult to modify over time. Accordingly, established self-care routines in this group may require additional or more tailored educational strategies to support meaningful behavioral change [33]. Despite improvements in self-care skills, no corresponding changes in glycemic control were observed. Given that participants were included in up to one year post-diagnosis, many may have been in partial remission, potentially attenuating detectable changes in glycemic outcomes. Early treatment adjustments, such as changes in insulin therapy and the introduction of diabetes technology (e.g., insulin pumps), also occur during this period and may influence glycemic outcomes [34]. While this study did not observe any improvements in glycemic control, evidence suggests that accurate carbohydrate counting supports long-term glycemic management [10,11,12,13]. Moreover, a recent meta-analysis by Wiyono et al. reported greater reductions in HbA1c among children and adolescents receiving carbohydrate counting compared with control groups across nine trials [15]. Furthermore, diabetes self-care has been linked to improved glycemic control, better health outcomes, and improved quality of life [10,35,36]. A key finding in our study is that the newly diagnosed group reported greater confidence in carbohydrate counting and experienced it as less challenging, an encouraging indicator for future self-care capacity. A systematic review further supports the importance of diabetes self-care interventions that incorporate dietary knowledge to empower individuals with T1D in their self-care practices [37]. In relation to carbohydrate estimation accuracy skills, our results suggest that early dietary education is more effective in newly diagnosed families, while additional support may be needed for those with persistent glycemic challenges. These findings are consistent with those of Hawkes et al. who investigated the effect of a structured certified diabetes education program delivered during the first year after diagnosis, and reported improved glycemic control at 6, 12 and 18 months compared with a control group. However, this effect was not sustained at 24 months [38]. In our study glycemic control was assessed using TIR and postprandial glucose excursions to explore the dynamics of blood glucose fluctuations in greater detail. Although postprandial analyses did not yield statistically significant results, this study highlights the value of exploring glucose excursions beyond overall TIR. Analyzing glycemic variations throughout the day, particularly around main meals, may offer a more nuanced understanding of glucose patterns. Gaining such insight requires segmenting the day and assessing postprandial responses to specific meals. While overall TIR may appear stable across different periods, underlying challenges can vary substantially [35,39,40]. In this intervention, which focuses on carbohydrate counting, insulin dosing, and timing, we consider it essential to analyze these postprandial blood glucose curves specifically. Currently, there is no “golden standard” for examining meal-related fluctuations in sensor data where no information on meal bolus is given. Based on existing research, the detection threshold for identifying mealtime increased blood glucose in this study was defined as an increase of 0.03 mmol/L per minute sustained for 30 min [31,32]. However, this threshold may introduce some uncertainty into the analysis.

Group-based education programs have demonstrated positive effects on diabetes management, particularly by enhancing self-care skills and well-being [41,42]. The American Diabetes Association recommends family involvement in diabetes education [43], and previous studies have shown that such group-based education programs can improve quality of life [25,41,42]. Our recently published qualitative study exploring family perspectives and experiences with our group-based education program adds to the value of group settings in fostering peer-to-peer knowledge-sharing, emotional support, and for providing a safe and playful learning environment that actively engage children and adolescents in their own care [44].

A concern shared by many parents of children with diabetes is how to support the transition from parental dependence to greater adolescent independence in self-management [45,46]. Thus, in our study, parents and their children participated in the education program, engaging both jointly and separately in diabetes and diet-related exercises. Both groups reported increased confidence in the children’s carbohydrate counting skills, a key step in fostering greater independence. These findings underline the importance of involving families in education efforts.

This study has several limitations that warrant careful consideration. First, the small sample size and study design inherently limit the generalizability of the findings. Participation rates were particularly low in the SGC group, introducing a risk of selection bias and raising questions about the representativeness of the participants. The inclusion of two relatively small subgroups with distinct challenges further increased variability, particularly affecting the robustness of conclusions for the SGC group. A major limitation of this study is the absence of a control group. The non-controlled pre–post design limits causal inference, as observed changes may reflect external influences, natural variation over time, or individual differences rather than the intervention itself. This reduces the internal validity of the findings. Small-sample studies are also prone to greater variability in individual responses, which may skew results and lead to misleading conclusions. Moreover, small studies tend to overestimate effect sizes while lacking the statistical power to detect subtle, yet clinically relevant, effects [47]. Nevertheless, the observation that carbohydrate counting skills improved in the ND group but not in the SGC group suggests that these improvements may reflect genuine learning rather than mere exposure. Another important limitation is the use of non-validated questionnaires to assess self-reported outcomes. While the instruments provided practical insight, their psychometric properties are unknown, which may affect the reliability and interpretability of the results. Future studies should consider employing validated measures to strengthen confidence in the findings. Furthermore, an important limitation is the potential for social desirability bias, as the investigator also served as the educator. This dual role may have influenced participants to report more favorable outcomes, particularly in self-reported measures of confidence and perceived difficulty. Response bias is also a concern. While 80% of children in the ND group demonstrated improved carbohydrate estimation accuracy at six months, this is based on only 15 responses, and overall follow-up rates were low for both groups. Respondents may represent a particularly motivated or capable subgroup, limiting generalizability. Additionally, differences in questionnaire formats for children and adults may have influenced response patterns: children used a 5-point Likert scale with emotive smileys, whereas adults used more neutral verbal anchors. This discrepancy could have affected the neutrality and interpretability of children’s responses. Finally, engaging children, adolescents, and families living with a chronic condition in research presents practical challenges. Competing demands and limited time often constrain families’ ability to participate fully in preparation and evaluation tasks. These factors must be considered when interpreting the study’s findings and designing future interventions in similar populations.

## 5. Conclusions

This exploratory study found that early, structured, interactive, play-based group education was associated with improvements in carbohydrate counting skills, mealtime insulin dosing, and self-care confidence among children, adolescents, and families with newly diagnosed T1D. In contrast, limited changes were observed among children and adolescents with suboptimally controlled T1D. Although no corresponding improvements in glycemic control were observed, the changes in self-care skills suggest potential short-term gains in diabetes self-management during the complex transitional phase following diagnosis. Further research in larger, controlled studies is needed to examine sustainability and long-term clinical impact.

## Figures and Tables

**Table 1:** Baseline characteristics.

	All (*n* = 77)	ND (*n* = 55)	SGC (*n* = 22)
Age, years	10 (±3)	9 (±3)	12 (±2)
Gender, females, *n* (%)	42 (55)	26 (47)	16 (73)
Diabetes duration, days	540 (±961)	115 (±84)	1605 (±1291)
BMI, kg/m^2^	19 (16, 20)	17 (16, 19)	21 (19, 22)
HbA1c, %/mmol/mol	8.7/72 (±29)	9.2/77 (±32)	7.8/62 (±18)
TIR, %	70 (±20)	74 (±16)	59 (±24)
Insulin pump, *n* (%)	24 (31)	7 (13)	17 (77)
MDI + sensor, *n* (%)	76 (99)	54 (98)	22 (100)

Data are presented as mean and SD, median with interquartile range (IQR) or frequencies with percentages in parenthesis. Abbreviations: BMI, body mass index; HbA1c, glycated hemoglobin; MDI, Multiple daily injections; ND, newly diagnosed group; SGC, suboptimal glycemic control group defined as >7.5% (58 mmol/mol) in HbA1c; SD, Standard deviation; TIR, time in range.

**Table 2:** Distribution of responses from all children and adolescents in the study based on the “Everyday Life” questionnaire.

	Baseline (*n* = 67)					End of Intervention (*n* = 42)							Six-Months Follow-Up (*n* = 18)						
Selected Questions from the “Everyday Life” Questionnaire—Children	Not at All	A Little/To a Lesser Extent	Some/To Some Extent	Quite a Lot/To a Great Extent	Extremely/To a Very Great Extent	Do Not Know	Not at All	A Little/To a Lesser Extent	Some/To Some Extent	Quite a Lot/To a Great Extent	Extremely/To a Very Great Extent	Do Not Know	*p*	Not at All	A Little/To a Lesser Extent	Some/To Some Extent	Quite a Lot/To a Great Extent	Extremely/To a Very Great Extent	*p*
Diabetes management																			
Can you count carbohydrates by yourself?	22%	15%	22%	19%	21%		12%	14%	21%	29%	24%		<0.001	0%	6%	22%	56%	17%	0.066
Are you able to dose your meal insulin by yourself?	9%	3%	10%	18%	60%		5%	0%	5%	14%	76%		0.005	0%	0%	0%	28%	72%	0.049
Dietary practice																			
Do you eat according to dietary recommendations?	8%	8%	11%	9%	65%		8%	10%	13%	10%	3%	58%	0.393	11%	11%	28%	17%	33%	0.095
Do you ever feel angry, irritated, or sad because of diabetes?	26%	29%	23%	15%	8%		25%	38%	15%	13%	10%		0.333	0%	50%	28%	17%	6%	0.013
Carbohydrate counting and perceptions																			
Is it difficult to count carbohydrates?	13%	36%	22%	9%	19%		17%	37%	20%	22%	5%		0.031	23%	36%	23%	5%	5%	0.905

Distribution of responses (in percentages) for selected questions (no. 3, 4, 8, 11, and 13) from the “Everyday Life” questionnaire. Data shown for both children and adolescents either newly diagnosed with T1D or with suboptimal glycemic control. All questions and responses are available in Supplementary Table S8.

**Table 3:** Distribution of responses from all parents in the study based on the “Everyday Life” questionnaire.

	Baseline (*n* = 72)						End of Intervention (*n* = 44)						*p*	Six-Months Follow-Up (*n* = 17)						*p*
Selected Questions from the “Everyday Life” Questionnaire—Parents	Not at All (1)	A Little/To a Lesser Extent (2)	Some/To Some Extent (3)	Quite a Lot/To a Great Extent (4)	Extremely/To a Very Great Extent (5)	Do Not Know (6)	Not at All (1)	A Little/To a Lesser Extent (2)	Some/To Some Extent (3)	Quite a Lot/To a Great Extent (4)	Extremely/To a Very Great Extent (5)	Do Not Know (6)		Not at All (1)	A little/To a Lesser Extent (2)	Some/To Some Extent (3)	Quite a Lot/To a Great Extent (4)	Extremely/To a Very Great Extent (5)	Do Not Know (6)	
Diabetes management																				
To what extent can your child count carbohydrates by itself?	22%	22%	26%	21%	8%		14%	23%	41%	16%	7%		0.049	10%	7%	40%	27%	17%		0.002
To what extent can your child calculate the amount of insulin needed for the food eaten?	24%	6%	25%	26%	19%		14%	22%	18%	29%	18%		0.051	10%	3%	14%	45%	28%		0.050
To what extent do you feel alone with diabetes?	17%	36%	31%	14%	3%		9%	36%	38%	16%	2%		0.221	10%	45%	28%	14%	3%		0.477
Dietary practice																				
To what extent do you observe that your child eats healthily?	1%	13%	39%	47%	0%		4%	4%	47%	40%	4%		0.623	4%	14%	38%	45%	0%		0.056
To what extent do food or insulin cause conflicts?	21%	28%	35%	13%	4%		18%	41%	23%	14%	5%		0.618	21%	38%	28%	14%	0%		0.678
Carbohydrate counting																				
Do you find it difficult to count carbohydrates?	14%	44%	28%	7%	7%		22%	42%	22%	9%	4%		0.372	17%	62%	14%	4%	4%		0.067

Distribution of responses (in percentages) for selected questions (no. 3, 4, 8, 11, and 13) from the “Everyday Life” questionnaire. Data shown for both patients with a child with newly diagnosed T1D or suboptimal glycemic control. All questions and responses are available in Supplementary Table S9.

## Data Availability

The data that support the findings of this study are available on request from the corresponding author. The data are not publicly available due to privacy or ethical restrictions.

## Supplementary Materials

The following supporting information can be downloaded at: https://www.mdpi.com/article/10.3390/nu18050790/s1, File S1: Questionnaire 1; “Everyday life—Parents”: provided to children and parents after the first education session, at the end of education course, and at six-months follow-up. File S2: Questionnaire 1; “Everyday life—Children”: provided to children and parents after the first education session, at the end of education course, and at six-months follow-up. File S3: Questionnaire 2: “Carbohydrate estimation”: provided to children and parents after the first education session, at the end of education course, and at six-months follow-up. File S4: Evaluation form: provided to children and parents after the education course. The responses were anonymous. File S5: Table S5: Glucose sensor data incl. insulin pump; at baseline, end of intervention, and six-month follow-up. File S6: Table S6: Glucose sensor data excl. insulin pumps; adjustments for individuals who initiated insulin pump therapy during the intervention or follow-up period. File S7: Table S7: Mean and delta AUC values after breakfast, lunch, dinner for the SGC and ND group. File S8: Table S8: Questionnaire 1; “Everyday life—Children” responses: The total proportion of all responses. File S9: Table S9: Questionnaire 1; “Everyday life—Parents” responses: The total proportion of all responses.

## Abbreviations

The following abbreviations are used in this manuscript:

T1D	Type 1 diabetes
HbA1c	Hemoglobin A1c
SDCC	Steno Diabetes Center Copenhagen
MDI	Multiple daily injections
SP	Sundhedsplatformen (national electronic medical journal system)
TIR	Time-in-range
TAR	Time-above-range
TBR	Time-below-range
CV	Coefficient of variation
ND	Newly Diagnosed
SGC	Suboptimal Glycemic Control
FGM	Flash glucose monitoring
CGM	Continuous glucose monitoring
SD	Standard deviations
AUC	Area under the curve
BMI	Body mass index
