# Supplementary material for: Interactive and Play-Based Group Education Is Associated with Improvements in Carbohydrate Counting Skills and Self-Care Confidence in Children and Adolescents with Type 1 Diabetes: An Exploratory Study
Source: Nutrients. 2026 Feb 27;18(5):790. doi: 10.3390/nu18050790 (PMC12987063; doi:10.3390/nu18050790)
Supplement: Supplementary file 1 [file nutrients-18-00790-s001.zip › nutrients-4139145-supplementary.pdf]

Supplementary files S1-S9

Supplementary file S1

Questionnaire 1: Diabetes in Everyday Life – Parents

To be completed by parents

Child's age ____________________

Child's gender ☐ Girl ☐ Boy ☐ Other

Participating parent's gender ☐ Woman ☐ Man ☐ Other

Date of child's diabetes diagnosis (year and month) _______________________

Date today: _______________________

Part 1: Diabetes management

- How much do food and insulin occupy your daily life:

☐ Not much ☐ A little ☐ Somewhat ☐ A lot ☐ Far too much

- To what extent does your child decide for themselves what to eat?

☐ Not at all ☐ To a small extent ☐ To some extent ☐ To a large extent ☐ To a very large extent

- To what extent can your child count carbohydrates themselves?

☐ Not at all ☐ To a small extent ☐ To some extent ☐ To a large extent ☐ To a very large extent

- To what extent can your child calculate insulin dosage for meals?

☐ Not at all ☐ To a small extent ☐ To some extent ☐ To a large extent ☐ To a very large extent

- To what extent do you feel alone with the diabetes management?

☐ Not at all ☐ To a small extent ☐ To some extent ☐ To a large extent ☐ To a very large extent

- To what extent do you experience having a negative relationship with food due to diabetes?

☐ Not at all ☐ To a small extent ☐ To some extent ☐ To a large extent ☐ To a very large extent

Part 2: Dietary practice

- To what extent do you think your child eats healthy?

☐ Not at all ☐ To a small extent ☐ To some extent ☐ To a large extent ☐ To a very large extent

- To what extent do you find it difficult to follow dietary recommendations (the 7 dietary guidelines)?

☐ Not at all ☐ To a small extent ☐ To some extent ☐ To a large extent ☐ To a very large extent ☐ Don't know

- To what extent do you find that following the dietary recommendations provides stable blood sugar?

☐ Not at all ☐ To a small extent ☐ To some extent ☐ To a large extent ☐ To a very large extent ☐ Don't know

- To what extent do you feel your child must eat differently than peers due to diabetes?

☐ Not at all ☐ To a small extent ☐ To some extent ☐ To a large extent ☐ To a very large extent

- To what extent are food and insulin a source of conflict?

☐ Not at all ☐ To a small extent ☐ To some extent ☐ To a large extent ☐ To a very large extent

- How adventurous (willing to try new things) is your child with food?

☐ Not much ☐ A little ☐ Somewhat ☐ Very ☐ Extremely

Part 3: Carbohydrate counting

- Do you find it difficult to count carbohydrates?

☐ Not at all ☐ To a small extent ☐ To some extent ☐ To a large extent ☐ To a very large extent

- Do you find it difficult to manage your child's blood sugar after meals?

☐ Not at all ☐ To a small extent ☐ To some extent ☐ To a large extent ☐ To a very large extent

- What do you use to count carbohydrates? (Check all that apply):

☐ Diabetes Association's app "Carbohydrates"

☐ DiAPPlo

☐ Nutritional declaration

☐ Internet search

☐ We know it by heart

☐ Nothing

☐ Other ________________

Supplementary file S2

Questionnaire 1: Diabetes in Everyday Life – Children/Adolescents

To be completed by children/adolescents

Everyday life

Your name: ____

How old are you? ______________________________

What is your sex/gender? ☐Girl ☐Boy ☐Other

When did you get diabetes? _________________________

Date today:_______________________________________

PART 1: DIABETES MANAGEMENT

- How much do food and insulin influence your everyday life?

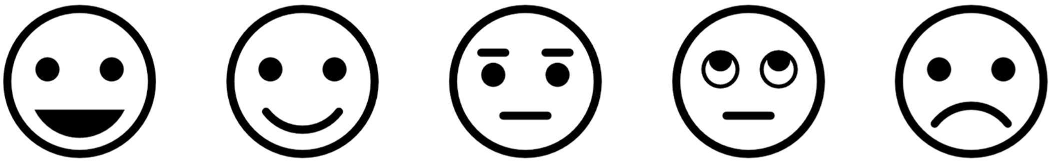

☐Not at all ☐A little ☐Some ☐Quite a lot ☐Extremely

- Are you able to manage what you want to eat?

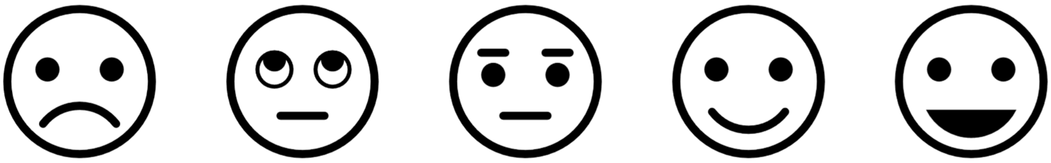

☐Not at all ☐A little ☐Some ☐Quite a lot ☐Extremely

- Can you count carbohydrates by yourself?

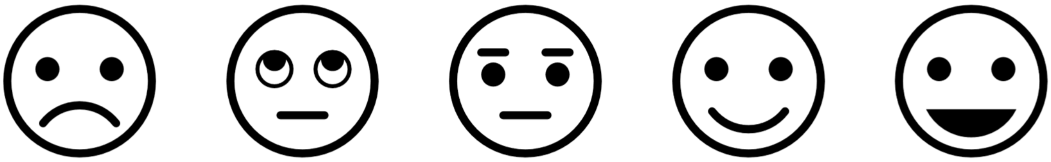

☐Not at all ☐A little ☐Some ☐Quite a lot ☐Extremely

- Can you dose your meal insulin by yourself?

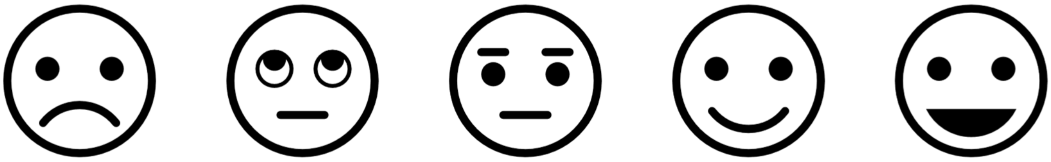

☐Not at all ☐A little ☐Some ☐Quite a lot ☐Extremely

- Do you feel alone with diabetes?

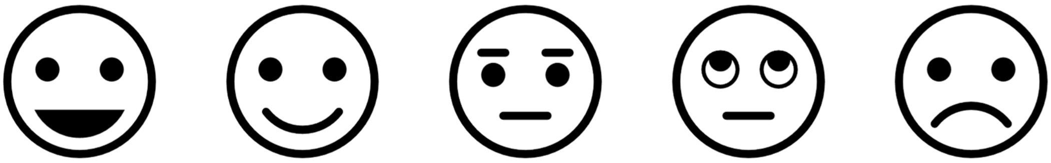

☐Not at all ☐A little ☐Some ☐Quite a lot ☐Extremely

- Do you have a negative relationship with food because of diabetes?

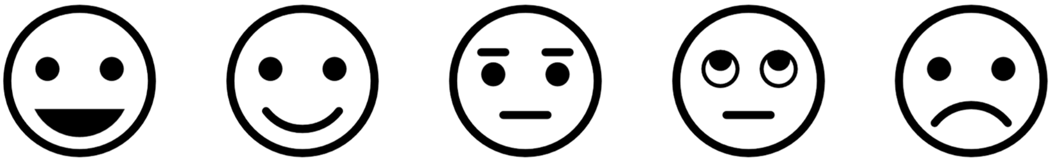

☐Not at all ☐A little ☐Some ☐Quite a lot ☐Extremely

Part 2: Dietary practice

- Do you think you eat healthily?

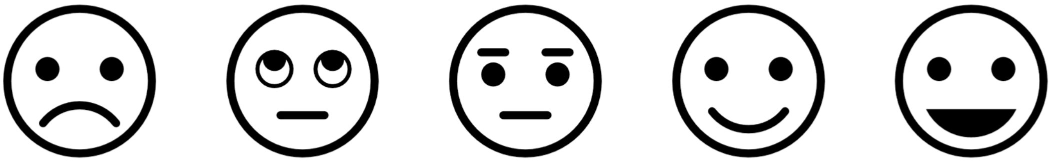

☐Not at all ☐A little ☐Some ☐Quite a lot ☐Extremely

- Do you eat according to dietary recommendations?

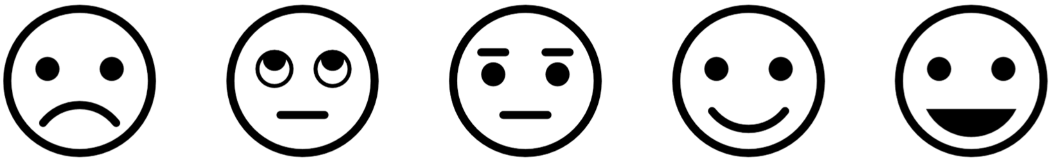

☐Not at all ☐A little ☐Some ☐Quite a lot ☐Extremely

- Do you find that following dietary guidelines helps maintain good blood sugar levels?

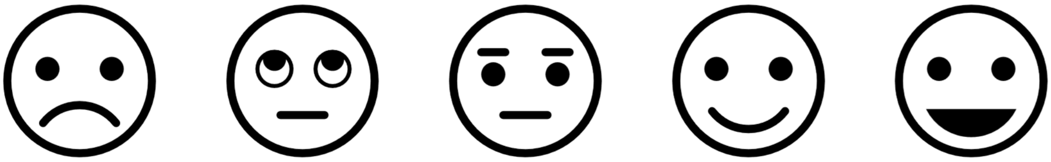

☐Not at all ☐A little ☐Some ☐Quite a lot ☐Extremely

- Do you feel that you have to eat differently than your friends because of diabetes?

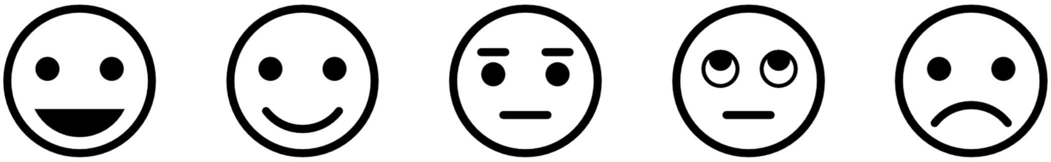

☐Not at all ☐A little ☐Some ☐Quite a lot ☐Extremely

- Do you ever feel angry, irritated, or sad because of diabetes?

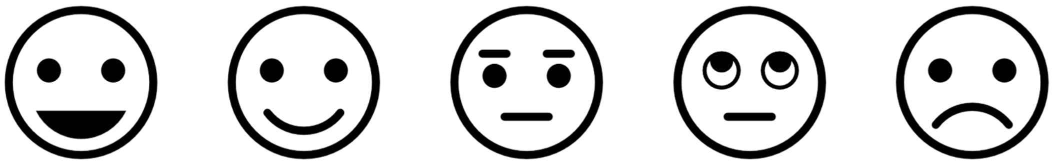

☐Not at all ☐A little ☐Some ☐Quite a lot ☐Extremely

- How willing are you to try new food?

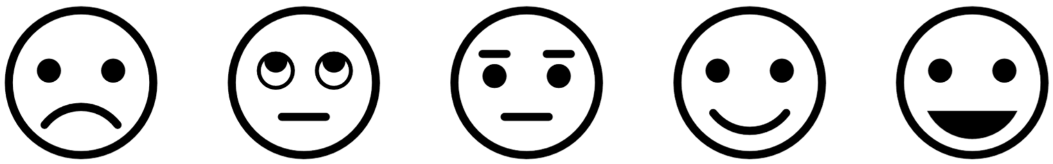

☐Not at all ☐A little ☐Some ☐Quite a lot ☐Extremely

Part 3: Carbohydrate Counting

- Is it difficult to count carbohydrates?

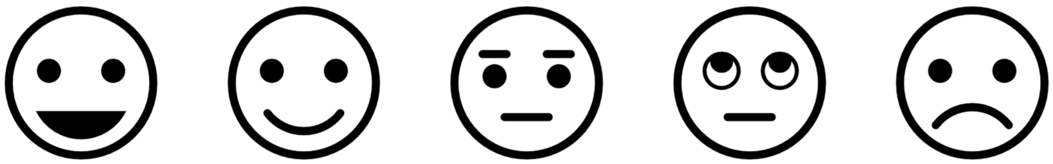

☐Not at all ☐A little ☐Some ☐Quite a lot ☐Extremely

- Is it difficult to manage your blood sugar after meals?

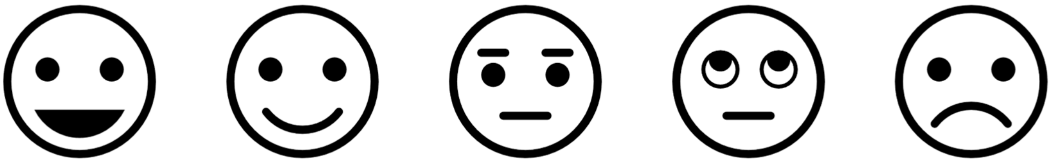

☐Not at all ☐A little ☐Some ☐Quite a lot ☐Extremely

- What do you use to count carbohydrates (Sæt gerne flere X):

☐ Diabetes Association’s app “Carbohydrates”

☐ DiAPPlo

☐ Nutritional declaration

☐ Internet search

☐ We know it by heart

☐ Nothing

☐ Other _______________

Supplementary file S3

Carbohydrate estimation test

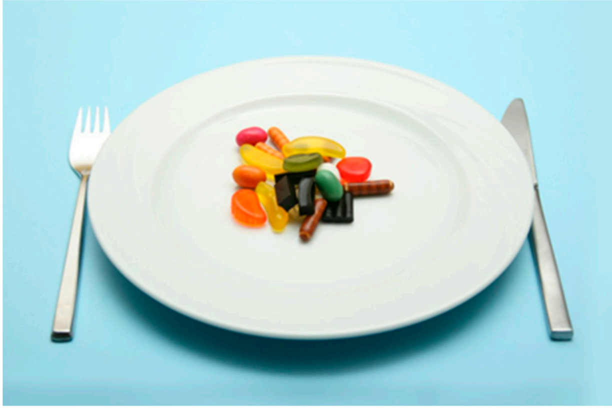
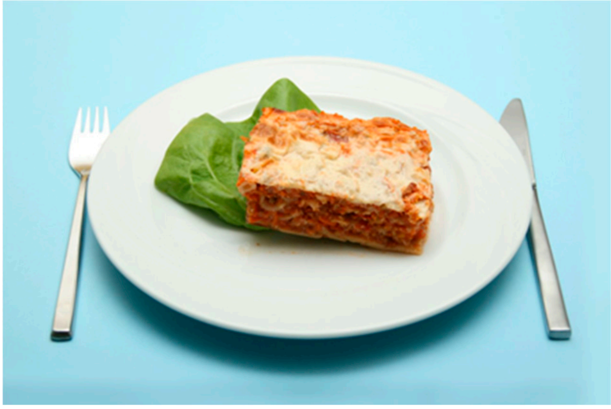
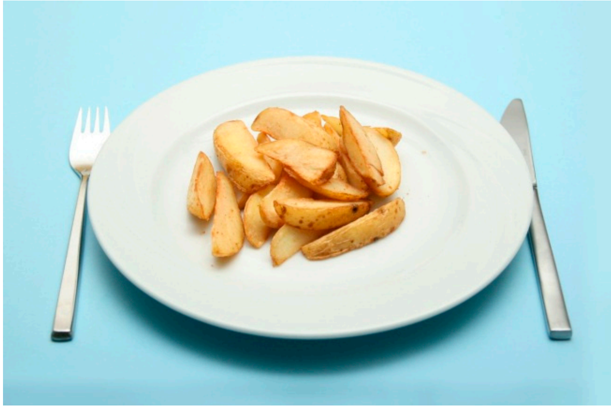
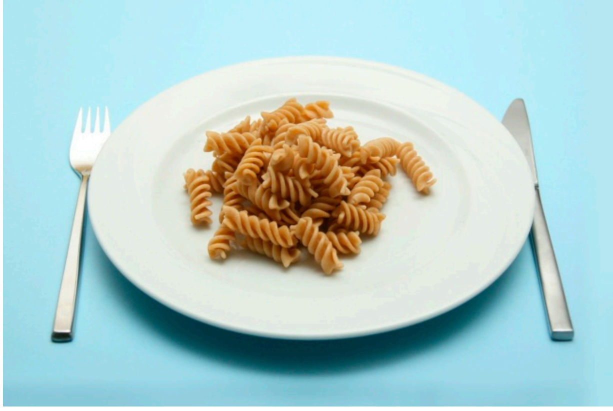
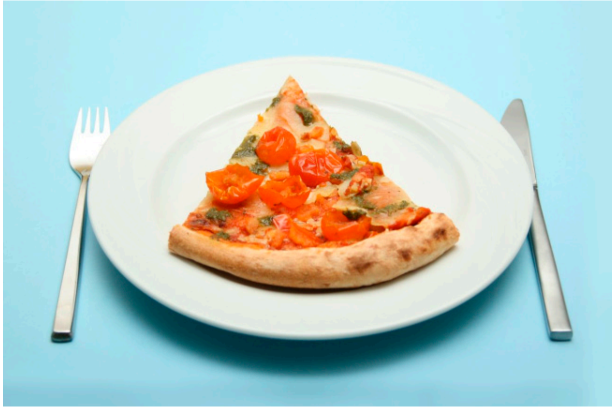
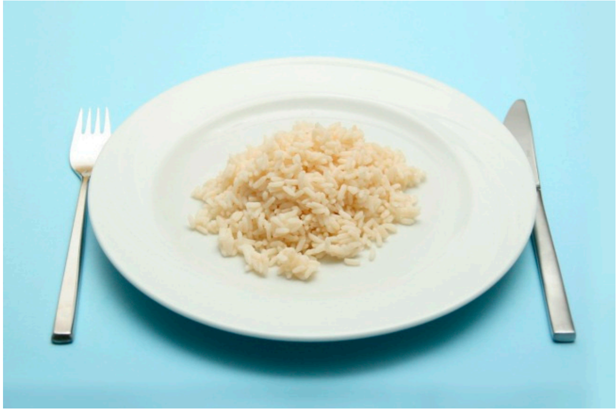

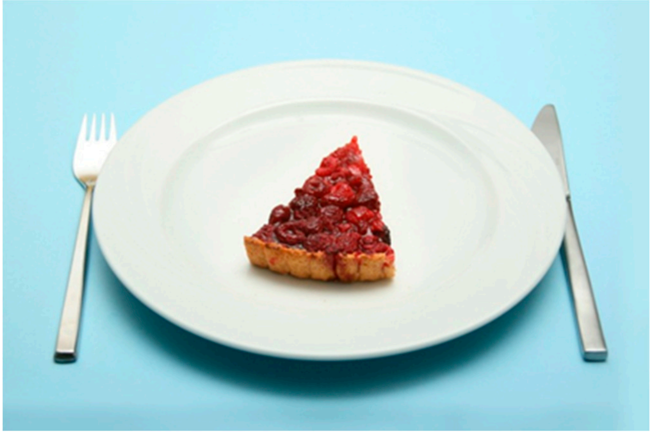
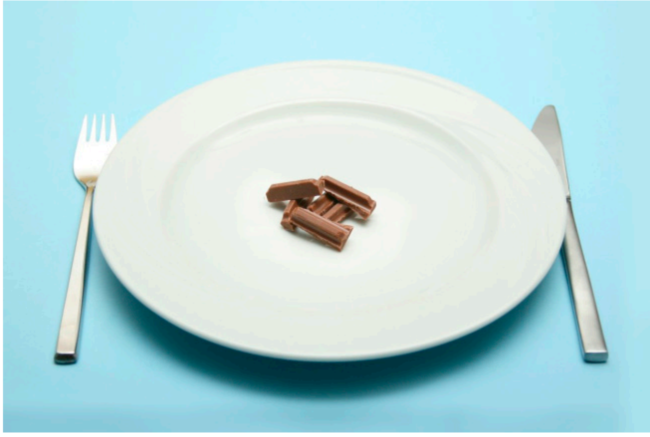
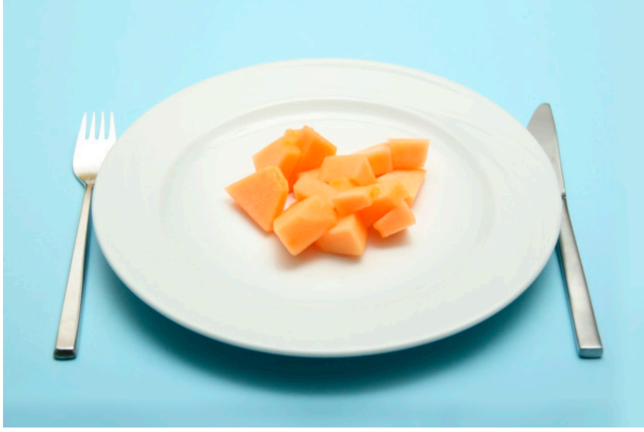
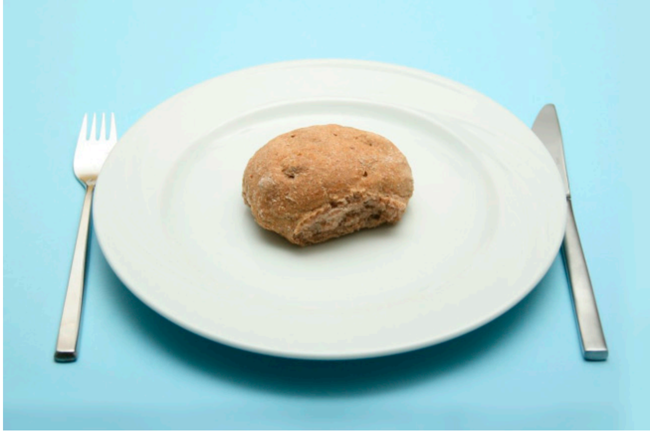
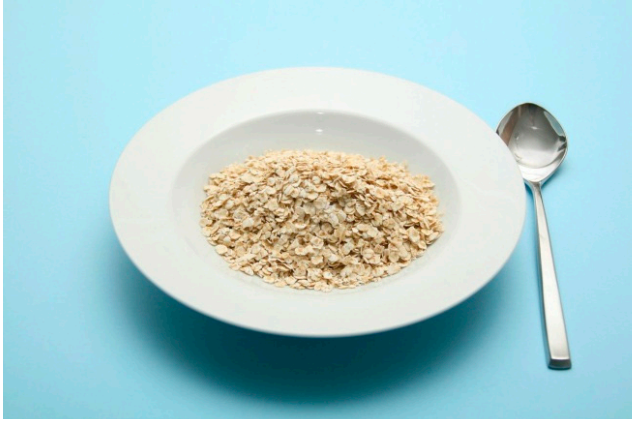
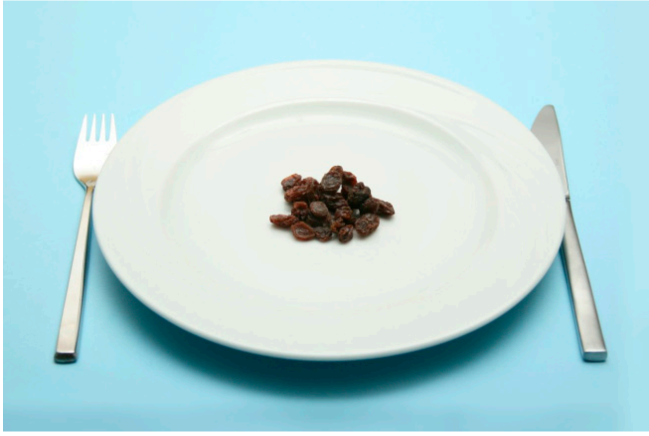

Supplementary file S4

Evaluation of the education program ”Food and Carbohydrates”

Child’s age: ____________________

Child’s gender: ☐Girl ☐Boy ☐Other

The sex of the participating parent ☐Female ☐Male ☐Other

Time of child's diabetes onset (year and month): _______________________

Registration

Why did you register for the course?______________________________

Who made you aware of the course?___________________________

Satisfaction

	Very satis-fied	Satisfied	Neu-tral	Dis-satis-fied	Very dis-satisfied
Content and activities at the first session “Food in Everyday Life”					
Content and activities at the second session “The Challenging Carbohy-drates”					
Content and activities at the third session “Celebration, Birthdays, and Holidays”					
Dietitians' way of involving and com-municating with your child					

	To a great extent	To some extent	To a small extent	Not at all
Do you feel it has been beneficial to be with other children/families?				
Was the time allocated for the course appropriate?				

Please elaborate on the above:

____

____

	To a great extent	To some extent	To a small extent	Not at all	Don't know
Do you think the course can replace individual dietitian consultations?					

What worked particularly well in the course?

____

____

____

What did not work in the course?

____

____

What do you think?

	To a great extent	To some extent	To a small extent	Not at all	Don't know
Your everyday challenges with food and blood sugar were included in the teaching?					
You helped set the agenda for the topics covered in the course?					
There was enough time to share experiences with other course participants?					

Content

	To a great extent	To some extent	To a small extent	Not at all	Don't know
Has it become easier to count carbohydrates after the course?					

	Yes	The same	No	Don't know
Is the child more involved in carbohydrate calculation now than before the course?				

	Yes	No	Don't know
Would you recommend the course to others?			

Do you have suggestions for improving the course?

____

Supplementary file S5

Table S5: Glucose sensor data incl insulin pump at baseline, end of intervention, and six-month follow-up.

	**Overall mean (SD)**					**Newly diagnosed diabetes (ND) mean (SD)**					**Suboptimal controlled T1D diabetes (SGC) mean (SD)**				
	Base-line	End of inter-vention.	*p*	Follow-up	*p*	Base-line	End of inter-vention	*p*	Follow-up	*p*	Base-line	End of inter-vention.	*p*	Follow-up	*p*
**TAR2, %**	10 (14) N=69	10 (13) N=55	0.743	11 (11) N=69	0.996	8 (12) N=54	7 (9) N=44	0.575	10 (12) N=54	0.547	16 (19) N=15	21 (17) N=11	0.544	13 (4) N=15	0.333
**TAR1, %**	17 (8) N=69	18 (8) N=55	0.393	21 (10) N=69	0.048	16 (8) N=54	17 (8) N=44	0.408	20 (11) N=54	0.028	22 (7) N=15	24 (3) N=11	0.816	23 (3) N=15	0.616
**TIR, %**	69 (14) N=71	70 (20) N=55	0.939	69 (13) N=69	0.973	73 (18) N=54	75 (16) N=44	0.535	71(14) N=54	0.253	57 (21) N=17	49 (22) N=11	0.311	61 (6) N=15	0.283
**TBR1, %**	2 (3) N=69	2 (3) N=55	0.746	2 (2) N=68	0.353	2 (3) N=54	2 (3) N=44	0.535	2 (1) N=53	0.505	2 (2) N=15	2 (2) N=11	0.616	2 (2) N=15	0.748
**TBR2, %**	0 (1) N=69	0 (0) N=55	0.410	0 (0) N=68	0.375	0 (1) N=54	0 (0) N=44	0.513	0 (0) N=53	0.387	0.0 (1) N=15	0 (0) N=11	0.608	0 (1) N=15	0.046
**CV, %**	35 (7) N=70	34 (7) N=56	0.179	36 (6) N=70	0.327	35 (7) N=55	33 (6) N=45	0.007	35 (6) N=55	0.793	36 (5) N=15	40 (5) N=11	0.032	40 (3) N=15	0.007
**Est HbA1c (mmol/mol)**	55 (13) N=72	54 (12) N=56	0.605	54 (8) N=70	0.684	53 (11) N=55	51 (9) N=45	0.450	54 (8) N=55	0.783	61 (16) N=17	66 (16) N=11	0.797	58 (4) N=15	0.334

Results are presented as mean and SD.
Abbreviations: Est HbA1c, sensor-estimated glycated hemoglobin; TAR1, time above range level 1 (10.1–13.9 mmol/l); TAR2, time above range level 2 (>13.9 mmol/l); TBR1, time below range, level 1 (3.0–3.8 mmol/l); TBR2, time below range, level 2 ()<3 mmol/l; TIR, time-in-range; CV%, coefficient of variation;
SD, standard deviation; mM, mmol/l; F.S, First session; L.S, Last session; F.U, Follow-up 6 months; ND, newly diagnosed diabetes; DD, suboptimal controlled T1D diabetes.

Supplementary file S6

Table S6: Glucose sensor data excl. insulin pumps: adjustments for individuals who initiated insulin pump therapy during the intervention or follow-up period.

	**Overall mean (SD)**					**Newly diagnosed diabetes (ND) mean (SD)**					**Suboptimal controlled T1D diabetes (SGC) mean (SD)**				
	Baseline	End of intervention.	*p*	Follow-up	*p*	Baseline	End of intervention.	*p*	Follow-up	*p*	End of intervention.	*p*	Follow-up	*p*	End of intervention.
**TAR2 (%) (>13.9 mM)**	9 (12) N=62	10 (13) N=49	0.685	13 (13) N=42	0.275	7 (8) N=48	8 (10) N=39	0.921	12 (15) N=29	0.063	16 (19) N=14	21 (18) N=10	0.373	14 (4) N=13	0.516
**TAR1 (%) (10.1–13.9mM)**	17 (8) N=62	19 (8) N=49	0.042	22 (11) N=42	0.043	16 (8) N=48	17 (9) N=39	0.018	22 (13)	0.055	22 (7) N=14	24 (7) N=10	0.614	24 (3)	0.546
**TIR (%) (3.9–10.0 mM)**	72 (18) N=62	70 (19) N=49	0.301	67 (13) N=42	0.295	76 (16) N=48	74 (16) N=39	0.360	96 (14)	0.101	59 (20) N=16	53 (17) N=10	0.614	60 (2)	0.584
**TBR1 (%) (3.0–3.8 mM)**	2 (3) N=62	2 (3) N=49	0.771	2 (2) N=42	0.469	2 (3) N=48	2 (3) N=39	0.569	2 (2)	0.497	2 (2) N=14	2 (2) N=10	0.4164	2 (2)	0.821
**TBR2 (%) (<3.0 mM)**	0 (1) N=62	0 (0) N=49	0.154	0 (0) N=42	0.136	0 (1) N=48	0 (0) N=39	0.271	0 (0)	0.426	0 (0) N=14	0 (0) N=10	0.672	0 (0)	0.046
**CV%**	35 (7) N=62	34 (7) N=49	0.396	37 (6) N=42	<0.001	33 (5) N=29	32 (6) N=21	0.414	36 (6) N=29	0.015	36 (5) N=14	40 (5) N=10	0.084	40 (3) N=13	0.013
**Est HbA1c (mmol/mol)**	53 (11) N=62	55 (13) N=49	0.552	55 (8) N=42	0.557	51 (8) N=48	52 (10)	0.848	54 (9) N=29	0.123	60 (16) N=14	66 (17) N=10	0.365	59 (4) N=13	0.538

Results are presented as mean ± SD
Abbreviations: Est HbA1c, sensor estimated glycated hemoglobin; TAR, time above range; TBR, time below range; TIR, time-in-range; CV%, coefficient of variation; SD, standard deviation; mM, mmol/l; F.S, First session; L.S, Last session; F.U, Follow-up 6 months; ND, newly diagnosed diabetes; DD, suboptimal controlled T1D diabetes.

Supplementary file S7

Table S7: Mean and delta AUC values after breakfast, lunch, dinner for the SGC and ND group

	AUC - SGC					***AUC - ND***				
	**Baseline (mean, mmol*h/l)**	**End of intervention (mean, mmol*h/l)**	***p***	**Follow-up (mean, mmol*h/l)**	***p***	**Baseline (mean, mmol*h/l)**	**End of intervention (mean, mmol*h/l)**	***p***	**Follow-up (mean, mmol*h/l)**	***p***
**Breakfast**	31.02 (12.76) N=10	35.66 (12.35) Δ + 4.64 N=6	0.49	26.36 (11.53) Δ - 4,66 N=7	0.45	26.82 (6.51) N=50	27.37 (7.20) Δ + 0.55 N=37	0.71	26.92 (5.53) Δ + 0.10 N=24	0.95
**Lunch**	35.43 (16.47) N=10	31.50 (7.64) Δ – 3.93 N=6	0.60	25.66 (4.82) Δ -9.77 N=7	0.15	24.53 (6.03) N=49	24.83 (5.25) Δ + 0.30 N=37	0.82	29.24 (9.37) Δ +4.41 N=29	0.009
**Dinner**	32.18 (9.13) N=9	40.15 (12.12) Δ + 7.97 N=6	0.20	30.54 (10.71) Δ – 1.64 N=8	0.74	27.35 (6.87) N=49	26.26 (5.53) Δ -1.09 N=36	0.44	30.61 (7.94) Δ +3.26 N=28	0.06

Results are presented as mean ± SD. Delta AUC values are based on values from baseline to end of intervention at one month, and from baseline to six-month follow-up. Abbreviations: AUC, Area under the curve; SGC, Suboptimal glucose control; ND: Newly Diagnosed.

Supplementary file S8

Table S8: Questionnaire 1; “Everyday life – Children” responses: The total proportion of all responses.

	Baseline (n=67)						End of Intervention (n=42)						*P*	Six-months follow-up (n=18)						*P*
Questions in “Every-day life” – Children.	**Not at all**	**A little / To a lesser extent**	**Some / To some extent**	**Quite a lot / to a great ex-**	**Extremely /to a very**	**Do not know**	**Not at all**	**A little / To a lesser extent**	**Some / To some extent**	**Quite a lot / to a great ex-**	**Extremely /to a very**	**Do not know**		**Not at all**	**A little / To a lesser extent**	**Some / To some extent**	**Quite a lot / to a great ex-**	**Extremely /to a very**	**Do not know**	
Diabetes management																				
1: How much do food and insulin influence your everyday life?	10%	43%	31%	10%	5%		12%	43%	38%	5%	2%		0.884	11%	61%	22%	6%	0%		0.332
2: Are you able to manage what you want to eat?	2%	5%	24%	45%	25%		12%	0%	21%	38%	29%		0.710	0%	0%	39%	50%	11%		0.332
3: Can you count carbohydrates by yourself?	22%	15%	22%	19%	21%		12%	14%	21%	29%	24%		**<0.001**	0%	6%	22%	56%	17%		0.066
4: Can you dose your meal insulin by yourself?	9%	3%	10%	18%	60%		5%	0%	5%	14%	76%		**0.005**	0%	0%	0%	28%	72%		**0.049**
5: Do you feel alone with diabetes?	40%	21%	28%	8%	3%		33%	31%	26%	7%	2%		0.674	44%	39%	6%	11%	0%		1.000
6: Do you have a negative relationship with food because of diabetes?	48%	31%	15%	5%	2%		48%	36%	14%	2%	0%		1.000	61%	22%	17%	0%	0%		1.000

7: Do you think you eat healthily?	0%	8%	38%	39%	16%		0%	10%	28%	45%	18%		0.555	0%	6%	56%	33%	6%		0.718
Dietary habits																				
8: Do you eat according to dietary recommendations?	8%	8%	11%	9%	65%		8%	10%	13%	10%	3%	58%	0.393	11%	11%	28%	17%	33%		0.095
9: Do you find that following dietary guidelines helps maintain good blood sugar levels?	2%	3%	17%	11%	68%		5%	3%	8%	8%	0%	78%	1.000	0%	0%	22%	17%	0%	61%	0.814
10: Do you feel that you have to eat differently than your friends because of diabetes?	46%	22%	24%	6%	2%		63%	15%	13%	5%	5%		0.173	50%	33%	6%	11%	0%		0.854
11: Do you ever feel angry, irritated, or sad because of diabetes?	26%	29%	23%	15%	8%		25%	38%	15%	13%	10%		0.333	0%	50%	28%	17%	6%		**0.013**
Carbohydrates																				
12: How willing are you to try new food?	6%	24%	15%	33%	22%		7%	15%	17%	27%	34%		0.227	0%	23%	32%	18%	23%	5%	0.056
13: Is it difficult to count carbohydrates?	13%	36%	22%	9%	19%		17%	37%	20%	22%	5%		**0.031**	23%	36%	23%	5%	5%		0.905
14: Is it difficult to manage your blood sugar after meals?	5%	30%	36%	25%	5%		8%	25%	53%	10%	5%		0.118	23%	23%	41%	14%	0%		0.375

Distribution of responses (in percentages). Data shown for children and adolescents either newly diagnosed with T1D or with suboptimal glycemic control.

Supplementary file S9

Table S9: Questionnaire 1; "Everyday life – Parents" responses: The total proportion of all responses.

Questions in "Everyday life" – Parents.	Baseline (n=72)						End of Intervention (n=44)						*p*
	**Not at all (1)**	**A little / To a lesser extent**	**Some / To some extent**	**Quite a lot / to a great ex-**	**Extremely /to a very**	**Do not know (6)**	**Not at all (1)**	**A little / To a lesser extent**	**Some / To some extent**	**Quite a lot / to a great ex-**	**Extremely /to a very**	**Do not know (6)**	
Diabetes management													
1: How much do food and insulin influence your everyday life?	1%	17%	33%	40%	8%		2%	16%	36%	39%	7%		0.700
2: To what extent is your child able to manage what it wants to eat?	3%	11%	33%	41%	11%		5%	5%	41%	36%	14%		0.627
3: To what extent can your child count carbohydrates by itself?	22%	22%	26%	21%	8%		14%	23%	41%	16%	7%		**0.049**
4: To what extent can your child calculate the amount of insulin needed for the food eaten?	24%	6%	25%	26%	19%		14%	22%	18%	29%	18%		0.051
5: To what extent do you feel alone with diabetes?	17%	36%	31%	14%	3%		9%	36%	38%	16%	2%		0.221
6: To what extent do you experience having a negative relationship with	29%	36%	26%	8%	0%		27%	47%	22%	5%	0%		0.860

Questions in "Everyday life" – Parents.	Six-months Follow-up (n=17)						*p*
	**Not at all (1)**	**A little / To a lesser extent**	**Some / To some extent**	**Quite a lot / to a great ex-**	**Extremely /to a very**	**Do not know (6)**	
Diabetes management							
1: How much do food and insulin influence your everyday life?	3%	30%	37%	27%	3%		0.433
2: To what extent is your child able to manage what it wants to eat?	0%	3%	30%	57%	10%		0.232
3: To what extent can your child count carbohydrates by itself?	10%	7%	40%	27%	17%		**0.002**
4: To what extent can your child calculate the amount of insulin needed for the food eaten?	10%	3%	14%	45%	28%		0.050
5: To what extent do you feel alone with diabetes?	10%	45%	28%	14%	3%		0.477
6: To what extent do you experience having a negative relationship with	24%	41%	24%	7%	3%		0.199

food due to diabetes?																				
7: To what extent do you observe that your child eats healthily?	1%	13%	39%	47%	0%		4%	4%	47%	40%	4%		0.623	4%	14%	38%	45%	0%		0.056
Dietary habits																				
8: To what extent do you find the dietary official dietary guidelines difficult to follow?	10%	26%	33%	10%	6%	15%	4%	22%	53%	9%	4%	7%	0.070	3%	24%	59%	7%	3%	3%	0.557
9: To what extent do you experience that following the official dietary guidelines helps maintain a more stable blood sugar level?	3%	1%	21%	33%	10%	32%	0%	7%	29%	22%	13%	29%	1.000	0%	7%	17%	34%	14%	28%	0.752
10: To what extent do you find that your child has to eat differently than their peers because of diabetes?	26%	36%	29%	7%	1%		22%	44%	13%	18%	2%		0.456	35%	31%	28%	3%	3%		0.345
11: To what extent do food and insulin cause conflicts?	21%	28%	35%	13%	4%		18%	41%	23%	14%	5%		0.618	21%	38%	28%	14%	0%		0.678
Carbohydrates																				
12: How willing is your child when it comes to trying new foods?	14%	23%	28%	31%	4%		11%	16%	34%	39%	0%		0.225	10%	28%	35%	21%	7%		0.802
13: Do you find it difficult to count carbohydrates?	14%	44%	28%	7%	7%		22%	42%	22%	9%	4%		0.372	17%	62%	14%	4%	4%		0.067

14: Do you find it difficult to manage your child's blood sugar after meals?	0%	25%	56%	18%	1%	0%	29%	51%	16%	4%	0.420	0%	35%	48%	17%	0%	0.202

Distribution of responses (in percentages). Data shown for parents with a child with newly diagnosed T1D or suboptimal glycemic control.
